# Supplementary material for: Salvage of the 5-deoxyribose byproduct of radical SAM enzymes
Source: Nat Commun. 2018 Aug 6;9:3105. doi: 10.1038/s41467-018-05589-4 (PMC6079011; doi:10.1038/s41467-018-05589-4)
Supplement: Supplementary file 1 — Supplementary Information [file 41467_2018_5589_MOESM1_ESM.pdf]

Supplementary Information

Salvage of the 5-deoxyribose byproduct of radical SAM enzymes

Beaudoin et al.

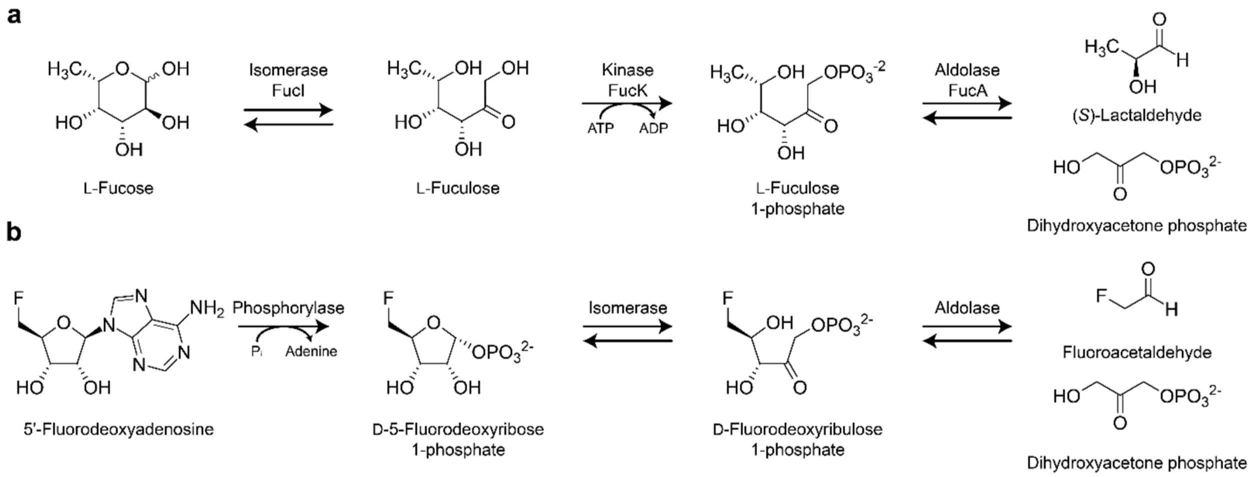

Supplementary Figure 1 | Known routes of L-fucose and 5'-fluorodeoxyadenosine metabolism. (**a**) The catabolism of L-fucose proceeds through an isomerase, a kinase, and an aldolase to give DHAP plus (*S*)-lactaldehyde. L-Rhamnose is metabolized via an analogous route. (**b**) In *Streptomyces cattleya*, 5′-fluorodeoxyadenosine is metabolized to 5-fluorodeoxyribose 1-phosphate by a phosphorylase, which is then acted upon by an isomerase and an aldolase to give DHAP plus fluoroacetaldehyde. Note that the phosphorylation and isomerase steps come in different order in the metabolism of L-fucose and 5'-fluorodeoxyadenosine.

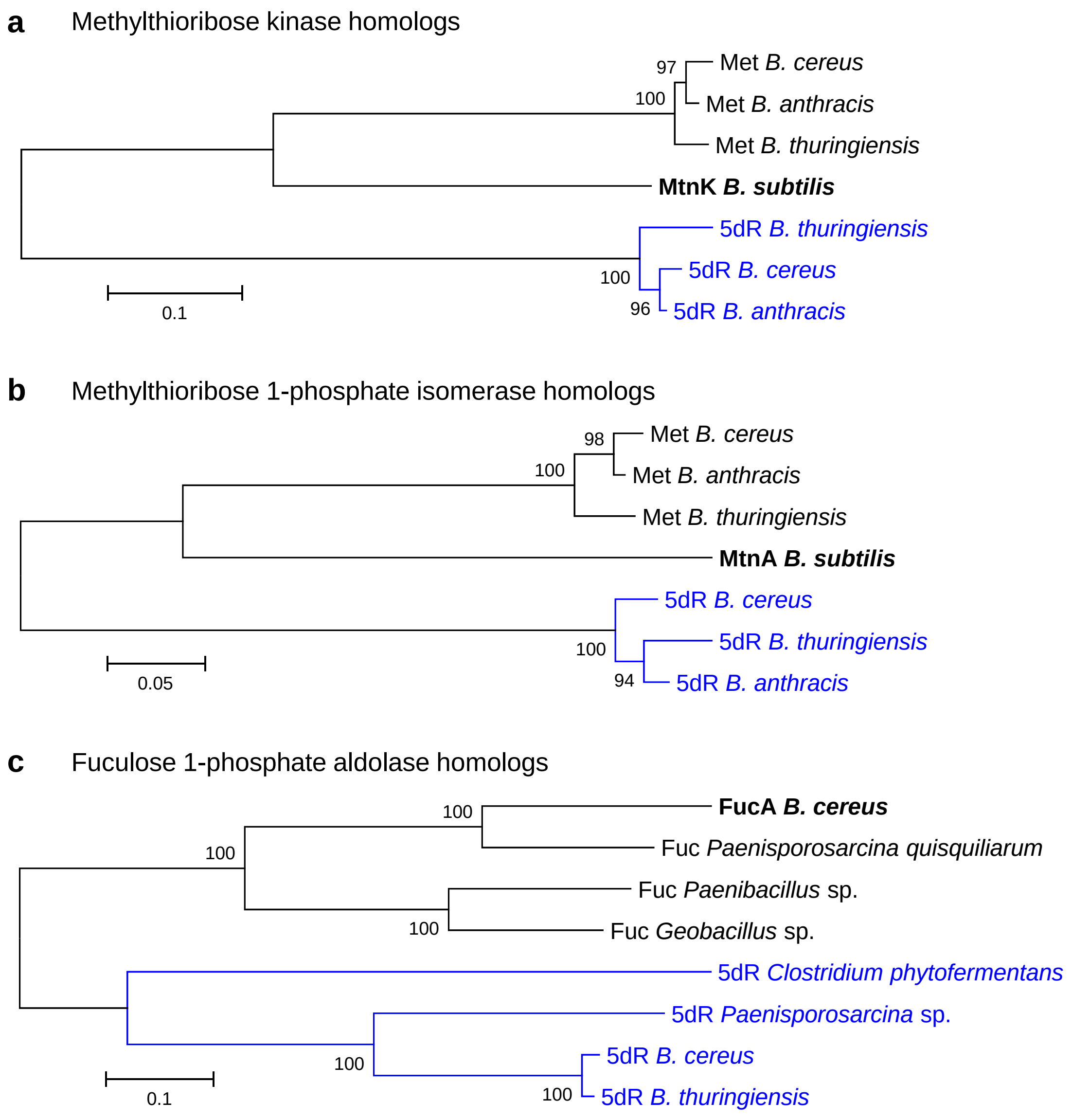

Supplementary Figure 2 | The kinase, isomerase, and aldolase encoded by clustered genes in Firmicutes are paralogs of the canonical enzymes of methionine salvage and fucose metabolism. Methylthioribose kinase-like, methylthioribose 1-phosphate isomerase-like, and fuculose 1-phosphate aldolase-like sequences from predicted 5-deoxyribose metabolism clusters (5dR) and from methionine salvage gene clusters (Met) or fucose metabolism clusters (Fuc) were aligned by ClustalW. Phylogenetic trees were constructed by the neighbor-joining method; bootstrap values (1,000 replicates) are next to nodes. Evolutionary distances are in units of the number of amino acid substitutions per site. (**a**) 5-Methylthioribose kinase homologs; the experimentally validated 5-methylthioribose kinase MtnK from *B. subtilis*[14] is included for comparison. (**b**) Methylthioribose 1-phosphate isomerase homologs; the validated methylthioribose 1-phosphate isomerase MtnA from *B. subtilis*[14] is included. (**c**) Fuculose 1-phosphate aldolase homologs; the validated fuculose 1-phosphate aldolase FucA from *B. cereus*[16] is included.

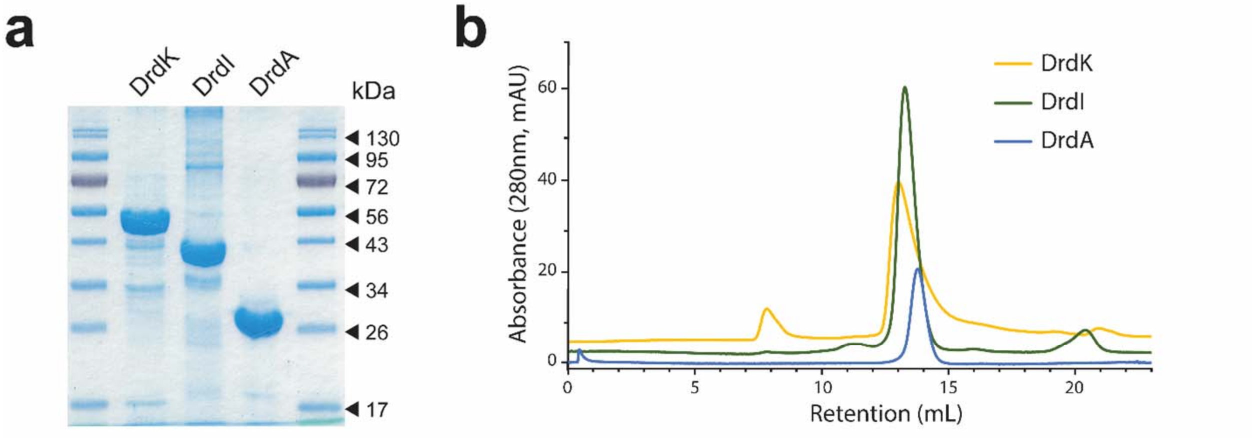

c

Protein	Calculated monomer mass (kDa)	Estimated native mass (kDa)	Ratio
DrdK	49.58	84.33	1.70
DrdI	40.50	76.67	1.89
DrdA	24.91	64.89	2.60

Supplementary Figure 3 | Characterization of recombinant *B. thuringiensis* DrdK, DrdI, and DrdA. (**a**) Coomassie-stained SDS-PAGE gel of proteins isolated by Ni^{2+}-affinity chromatography. (**b**) Superdex-200 gel filtration profiles of proteins isolated by Ni^{2+}-affinity chromatography. (**c**) Comparison of molecular masses calculated for the His-tagged monomers and native molecular masses estimated by gel filtration.

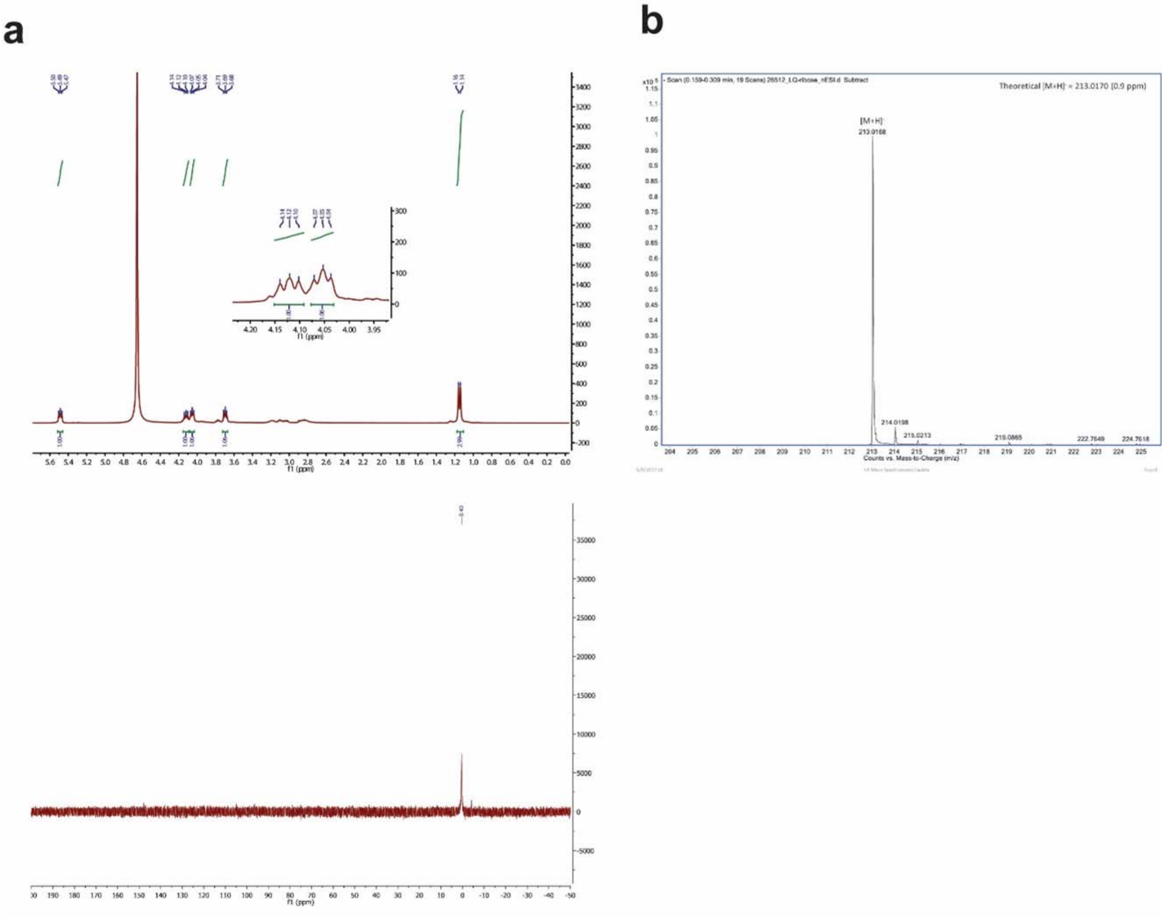

Supplementary Figure 4 | Spectral evidence that *B. thuringiensis* DrdK forms dR1P. (**a**) NMR of the 5-deoxyribose-1-phosphate (dR1P) reaction product of DrdK; ^{1}H (upper panel) and ^{31}P (lower panel) NMR spectra are shown, along with (**b**) high resolution mass spectral (ESI-TOF) analysis.

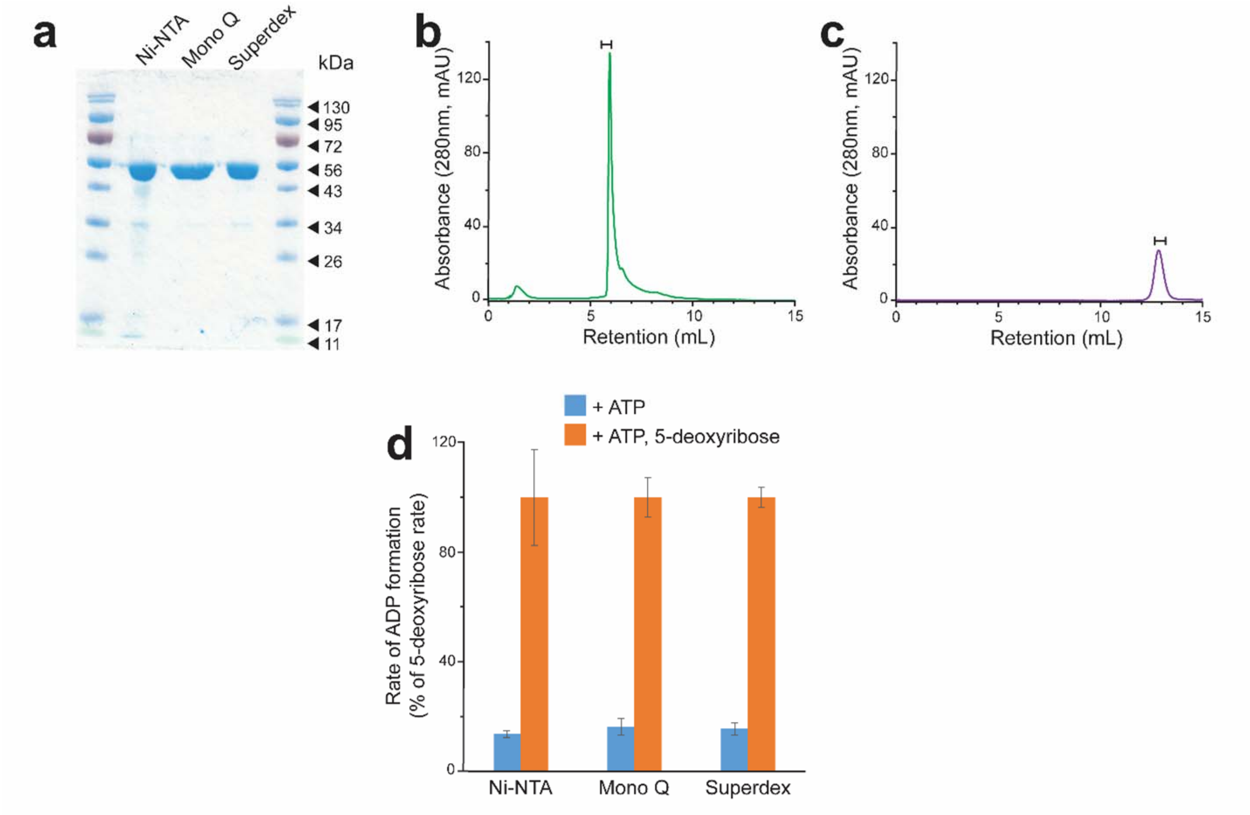

Supplementary Figure 5 | Evidence that *B. thuringiensis* DrdK has intrinsic ATPase activity. (**a**) Coomassie-stained SDS-PAGE gel of kinase protein isolated by Ni^{2+}-affinity chromatography and then further purified by successive anion exchange (Mono Q) and gel filtration (Superdex-200) steps. (**b**) Mono Q elution profile using a gradient of 0.050 - 1.0 M NaCl. (**c**) Superdex-200 gel elution profile. (**d**) Relative kinase (orange bars) and ATPase activities (blue bars) measured at successive purification steps using an assay that couples ADP formation to NADH oxidation. Data represent the mean of three replicates; error bars represent the s.d. The ratio of the activities remains constant after successive purification steps, showing that the ATPase activity is intrinsic and not due to contaminating enzymes with ATPase activity.

a **b**

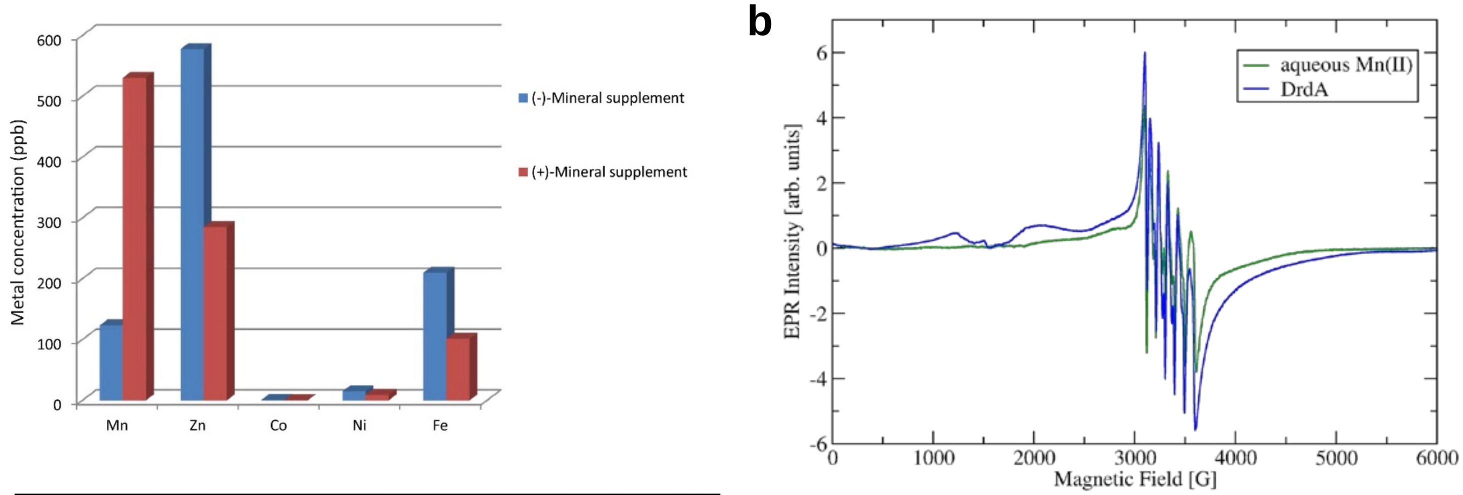

Sample	Mn	Zn	Co	Ni	Cu	Fe‡
(-)-Mineral supplement (MS)	123.3†	577.7	0.925	14.916	19.404	209.92
Metal/DrdA ratio	0.05	0.22	<0.001	0.006	0.007	0.09
(+)-Mineral supplement	530.36	284.67	0.573	8.807	29.807	101.27
Metal/DrdA ratio	0.23	0.11	<0.001	0.004	0.01	0.04

Supplementary Figure 6 | Analysis of the metal content of DrdA. Tag-free recombinant DrdA was produced in *E. coli* cells supplemented with trace minerals; the protein was purified by ammonium sulfate precipitation followed by anion exchange chromatography. (**a**) Protein metal content analysis of DrdA by inductively coupled plasma mass spectrometry (ICP-MS). (**b**) EPR spectrum of DrdA (blue trace) and aqueous Mn(II) in buffer at pH7 (green trace). The characteristic six-line signal at g ≈ 2 (3400 G) is very similar indicating the presence of unbound Mn(II) in the protein sample. However, clear differences are seen in both the overall line-width of the six-line signal which is broader for the protein sample as well as the appearance of low-field bands near 1300 G and 2000 G. This represents an additional Mn(II) species with a much larger magnitude of the fine structure (D parameter) than seen in hexaquo-Mn. It is indicative of protein-bound Mn(II)[28]. The weak signal at ~1500 G most likely represents Fe(III) in a rhombic environment. The experimental conditions for the EPR spectra were as follows. 1. Green trace: microwave frequency, 9.4008 GHz; microwave power, 2.00 mW; modulation frequency, 100 kHz; modulation amplitude, 5 G; conversion time per point, 40 ms; 1 scan; Bruker ER4122SHQE resonator; temperature, 6 K. 2. Blue trace: microwave frequency, 9.6454 GHz; microwave power, 0.317 mW; modulation frequency, 100 kHz; modulation amplitude, 10 G; conversion time per point, 40 ms; average of 2 scans; Bruker ER4116DM resonator; temperature, 6 K. After baseline correction he spectrum of the DrdA sample (blue trace) was shifted by 90 G to lower fields along the field axis to account for the difference in microwave frequency and its intensity reduced to 76% of the original for better comparison with the buffer sample (green trace). †Parts per billion. ‡Iron analysis was carried out separately, and has an inherently higher background due to instrument limit and error.

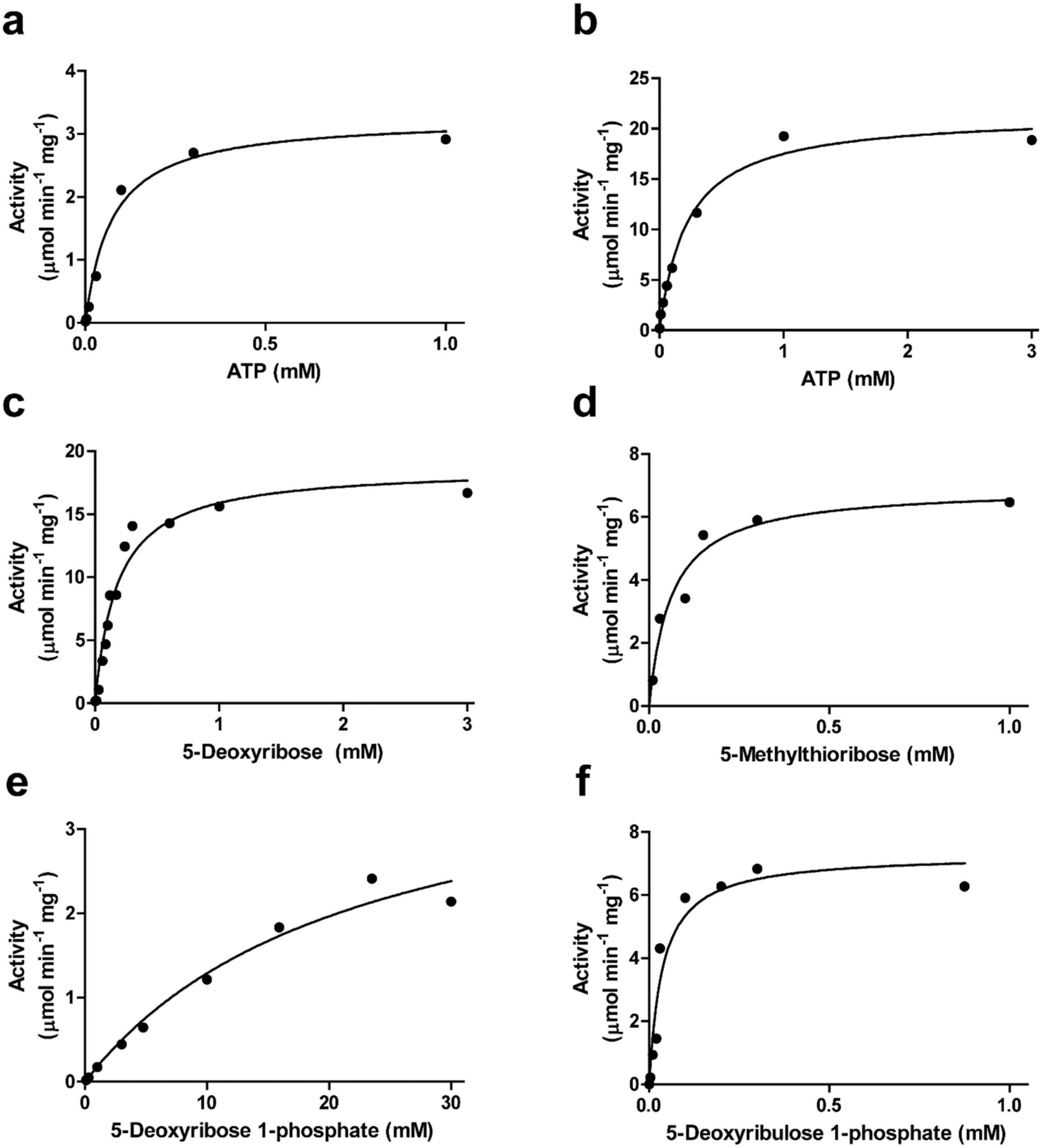

Supplementary Figure 7 | Primary data from kinetics experiments. (**a**,**b**) 5-Deoxyribose kinase (DrdK) Michaelis-Menten saturation curve for the determination of the K_m and k_{cat} for ATP consumption without 5-deoxyribose (**a**) or with 500 µM 5-deoxyribose (**b**). (**c**,**d**) Saturation curve for the phosphorylation of 5-deoxyribose (**c**) and 5-methylthioribose (**d**) with 1 mM ATP. (**e**,**f**) Saturation curve for 5-deoxyribose 1-phosphate isomerase (DrdI) (**e**) and 5-deoxyribulose 1-phosphate aldolase (DrdA) (**f**). Data are single representative experiments, from the set of three used to calculate the K_m and k_{cat} values of Table 1.

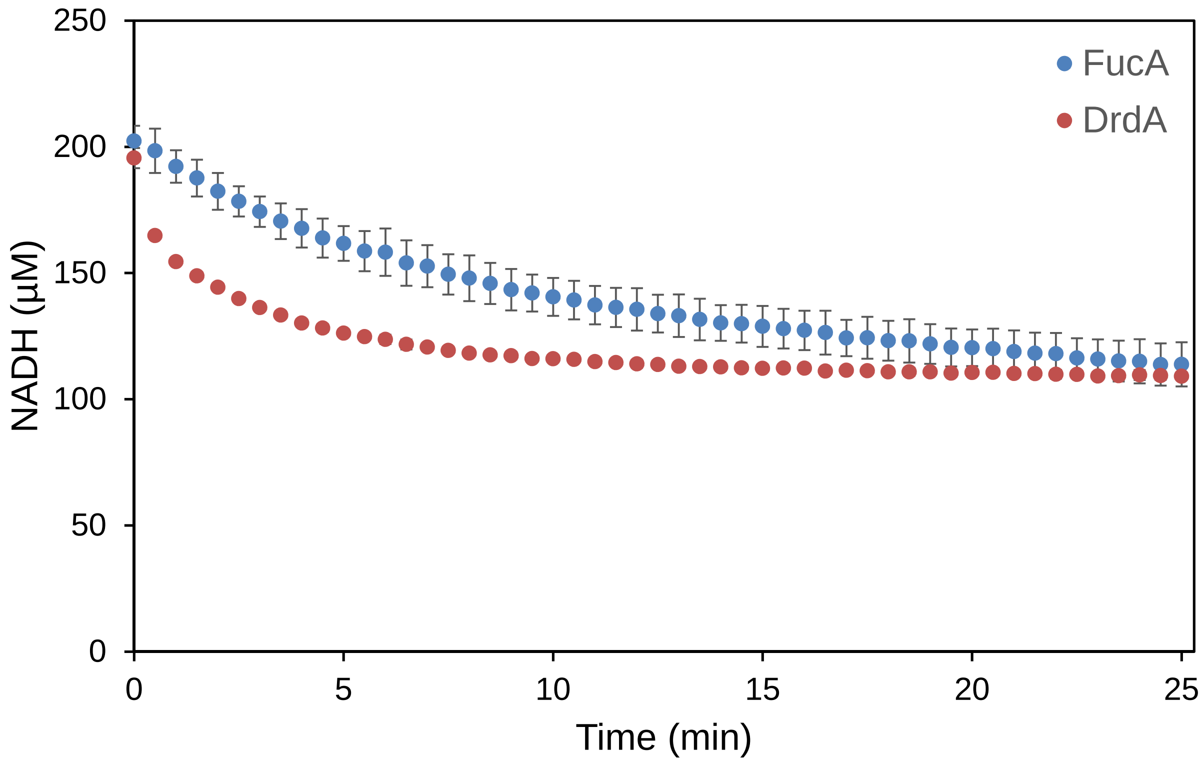

Supplementary Figure 8 | Use of *E. coli* fuculose 1-phosphate aldolase to investigate the configuration of the product formed by *B. thuringiensis* DrdA from DHAP and acetaldehyde. *E. coli* fuculose 1-phosphate aldolase (FucA) is known to be specific for (3*R*,4*R*)-2-keto-1-phosphosugars[27]. 5-Deoxyribulose 1-phosphate (90 µM) prepared from acetaldehyde and DHAP using the *B. thuringiensis* 5-deoxyribulose 1-phosphate aldolase (DrdA) was completely consumed by either this enzyme or FucA. DHAP formation from hydrolysis of 5-deoxyribulose 1-phosphate was measured via glycerol 3-phosphate dehydrogenase-catalyzed oxidation of NADH. This result indicates that DrdA has the same stereochemical preference as FucA. Data are the mean of three replicates; error bars are the s.d. Where no error bars are evident, they are smaller than the filled circle symbol.

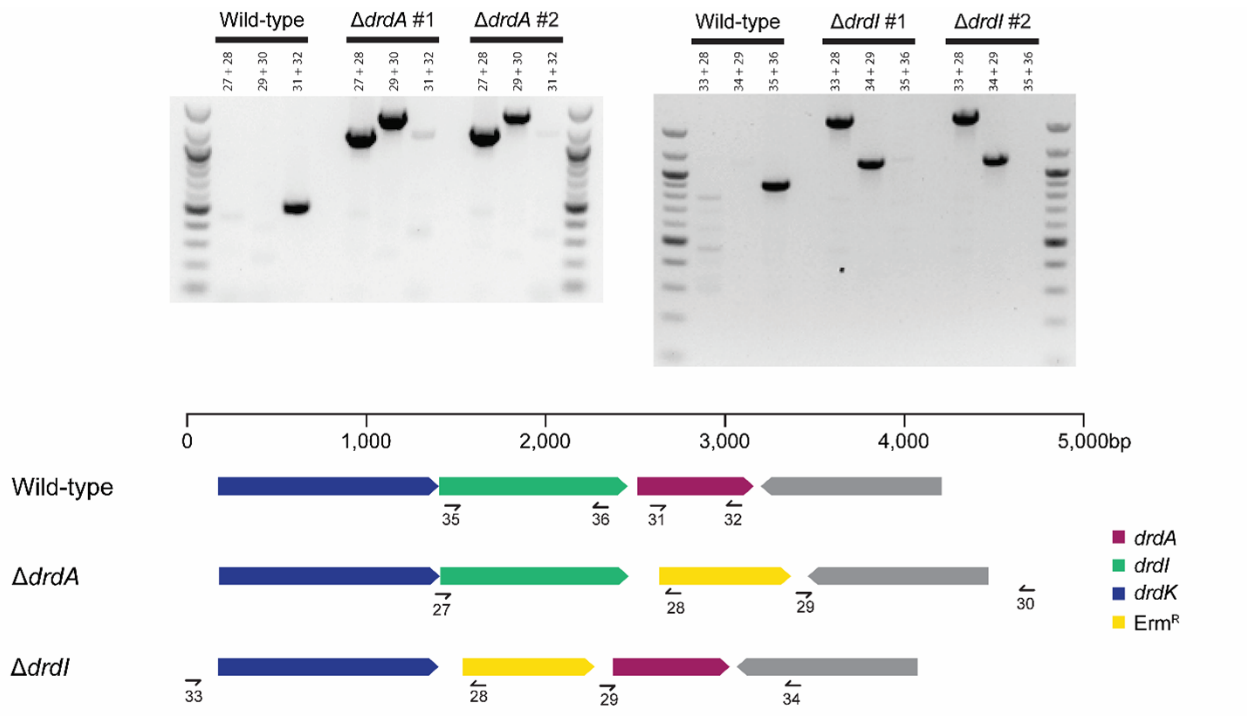

Supplementary Figure 9 | Validation of *B. thuringiensis drdI* and *drdA* deletants. Genomic DNA from two independent transformants of each deletant and the wild-type was used as template for PCR reactions. Primers 5′ and 3′ of the deletion sites (27 and 33, and 30 and 34, respectively) and primers at the 5′ and 3′ end of the Erm^R resistance cassette (28 and 29) were used to ensure the correct location of the insertion in the knockouts. Primers within *drdA* (31 and 32) or *drdI* (35 and 36) were used to verify that the gene was removed from the deletants, while remaining in the wild-type.

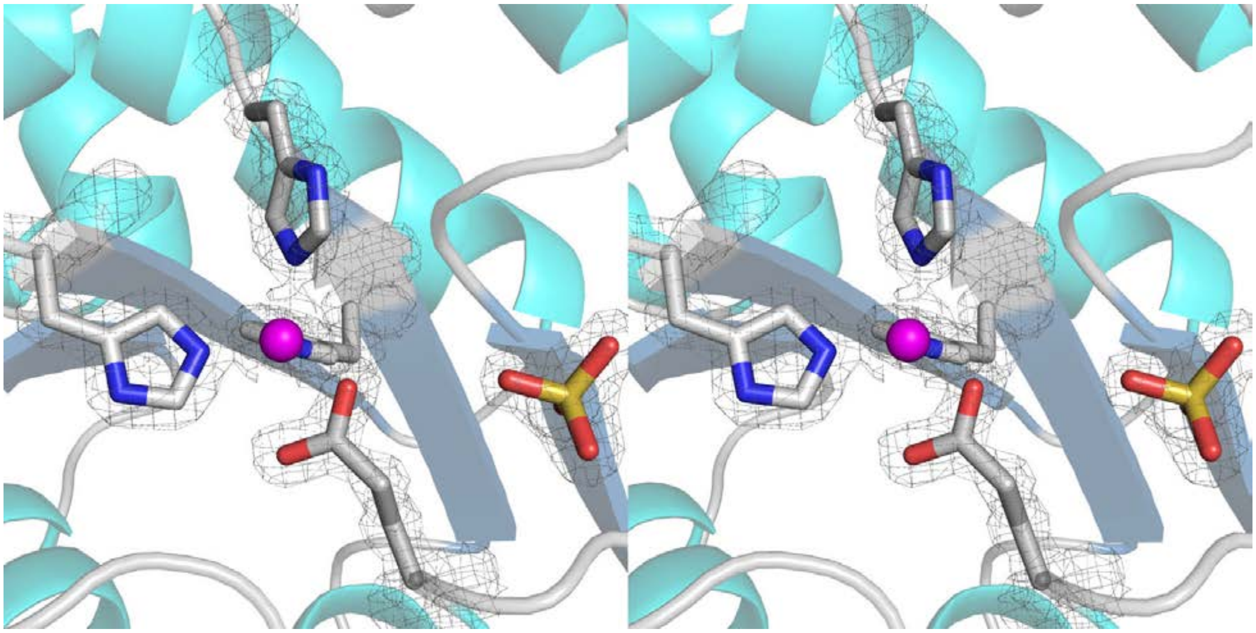

Supplementary Figure 10 | Stereoview of the electron density map at the DrdA active site. The side chains of metal coordinating residues (His95, His97, His157 and Glu76) are shown along with a bound inorganic sulfate. The protein backbone is represented in cartoon format. The 2Fo-Fc electron density map is contoured at 2.0 sigma.

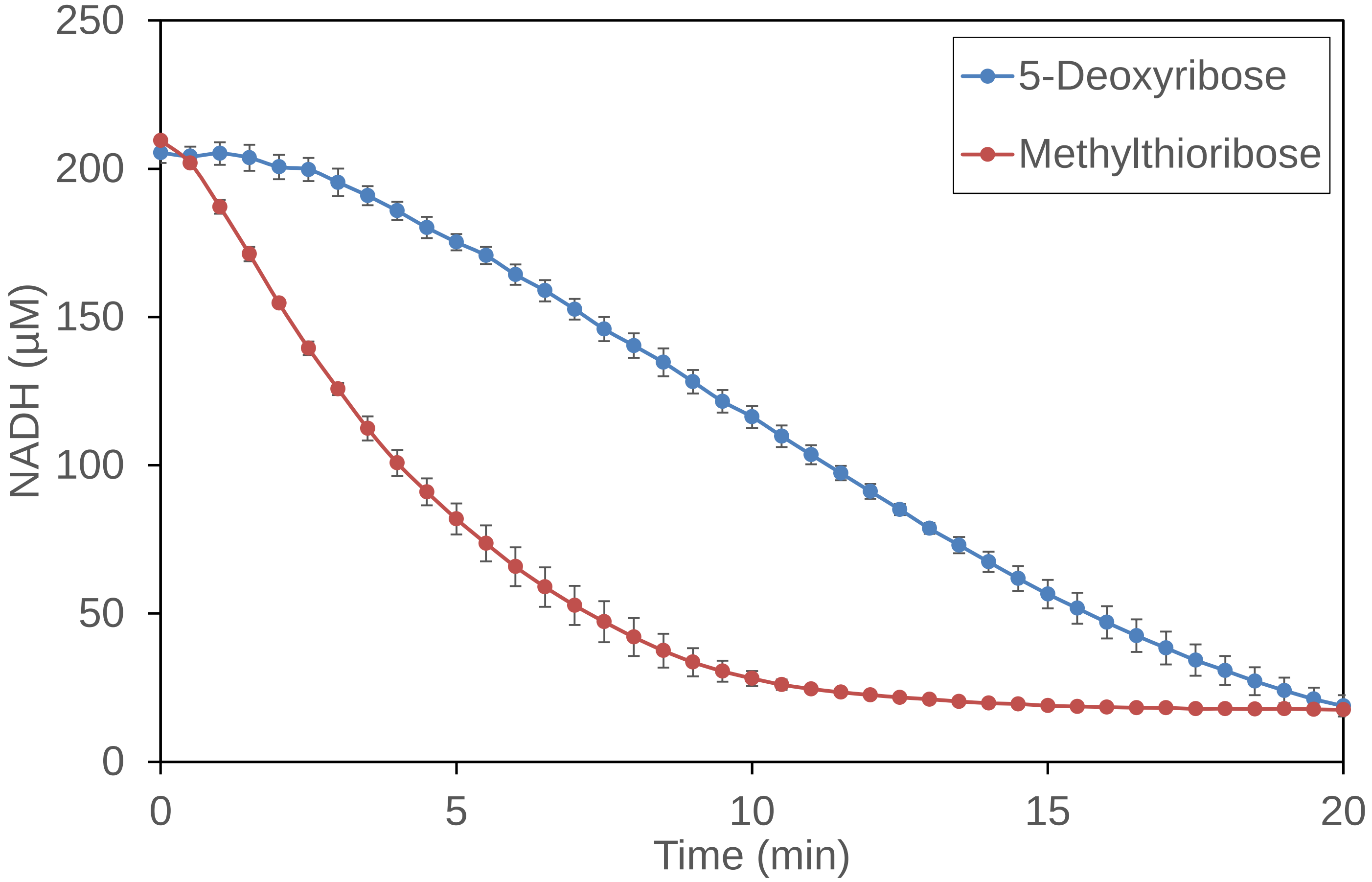

Supplementary Figure 11 | 5-Deoxyribose disposal enzymes are promiscuous and can convert 5-methylthioribose to DHAP and mercaptoacetaldehyde. Coupled assay of DHAP formation from 5-deoxyribose or 5-methylthioribose when DrdK, DrdI, DrdA, and ATP are present in the reaction. DHAP formation was detected via glycerol 3-phosphate dehydrogenase-catalyzed oxidation of NADH. Data are the mean of three replicates; error bars are the s.d.

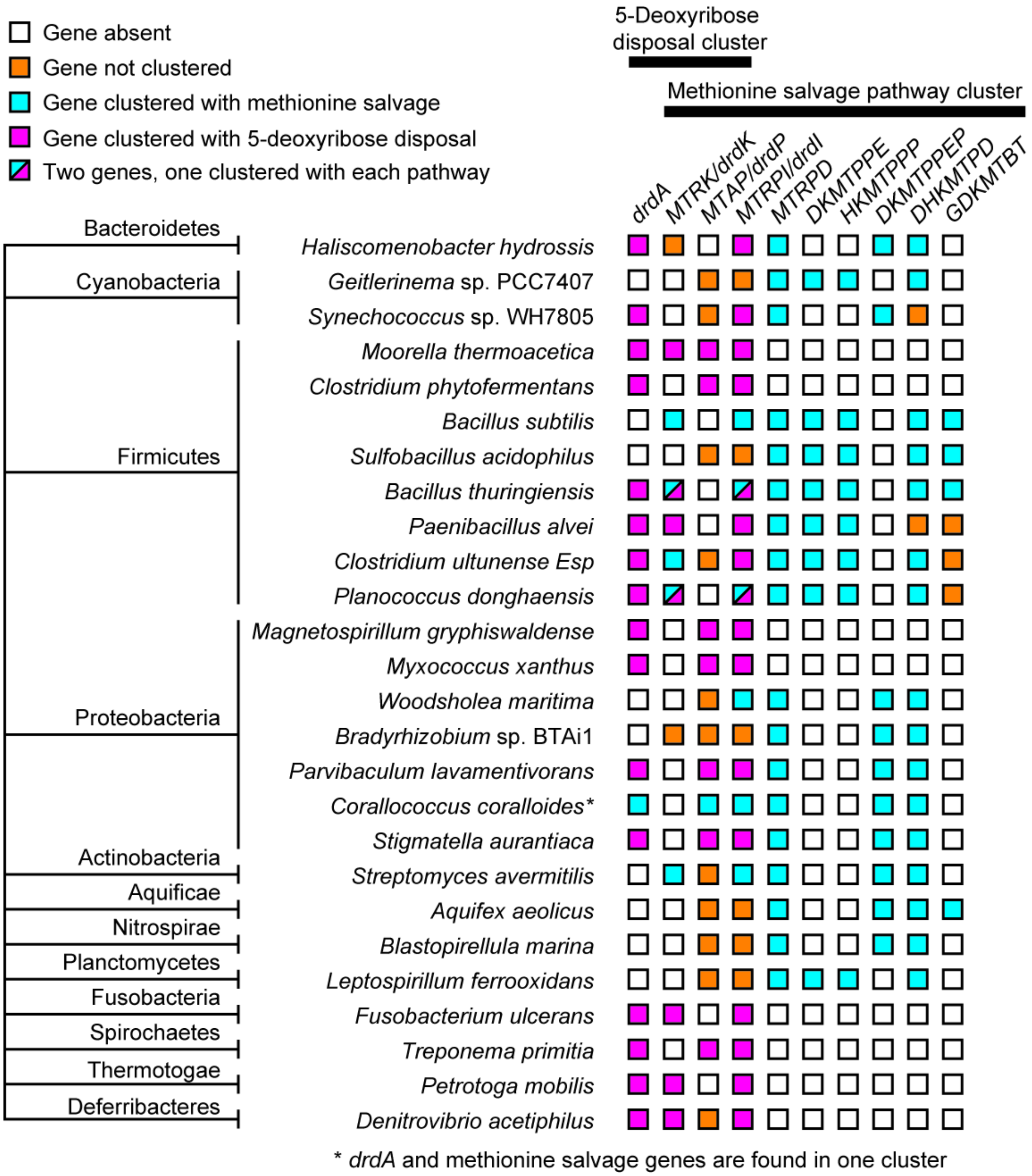

Supplementary Figure 12 | Comparative genomics points to dual roles for the kinase and isomerase in methionine salvage and 5-deoxyribose disposal in diverse bacteria. Organisms such as *Synechococcus* sp. WH7805 and *Haliscomenobacter hydrossis* have a single 5-methylthioribose 1-phosphate isomerase gene that clusters with a fuculose 1-phosphate aldolase, not with other methionine salvage pathway genes. In *Corallococcus coralloides*, an aldolase is clustered with all the methionine salvage pathway genes. Abbreviations: *drdA*, 5-deoxyribose disposal aldolase; *drdK*, 5-deoxyribose disposal kinase; *drdP*, 5-deoxyribose disposal phosphorylase; *drdI*, 5-deoxyribose disposal isomerase; MTRK, 5-methylthioribose kinase; MTAP, 5-methylthioadenosine phosphorylase; MTRPI, methylthioribose-1-phosphate isomerase; MTRPD, methylthioribulose-1-phosphate dehydratase; DKMTPPE, 2,3-diketo-5-methylthiopentyl-1-phosphate enolase; HKMTPPP, 2-hydroxy-3-keto-5-methylthiopentenyl-1-phosphate phosphatase; DKMTPPEP, 2,3-diketo-5-methylthiopentyl-1-phosphate enolase-phosphatase; DHKMTPD, 1,2-dihydroxy-3-keto-5-methylthiopentene dioxygenase; GDKMTBT, glutamine-dependent 2-keto-4-methylthiobutyrate transaminase.

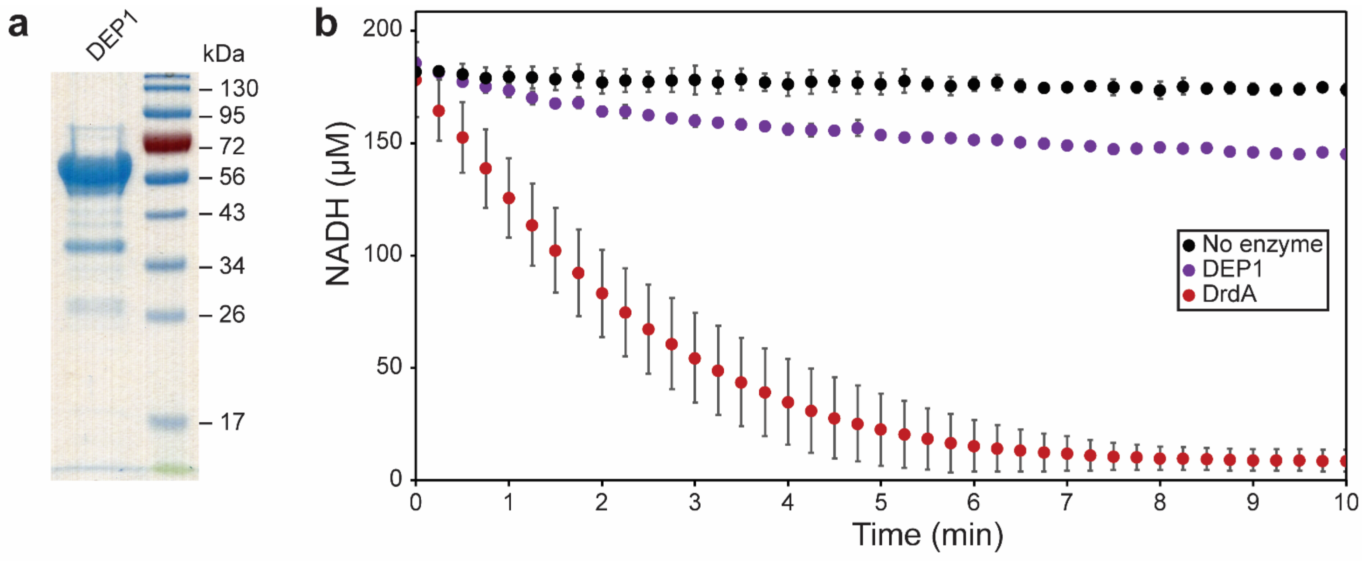

Supplementary Figure 13 | Aldolase activity of the *Arabidopsis* methionine salvage enzyme DEP1 against 5-deoxyribulose 1-phosphate. (**a**) Coomassie-stained SDS-PAGE gel of DEP1 (10 µg) isolated by Ni^{2+}-affinity chromatography. (**b**) The aldol cleavage of 5-deoxyribulose 1-phosphate (dRu1P; 500 µM) was measured by DHAP formation via glycerol 3-phosphate dehydrogenase-catalyzed oxidation of NADH. DEP1, a fusion enzyme containing class II aldolase and haloacid dehalogenase (HAD) domains, catalyzes the sequential dehydration, enolization, and dephosphorylation of methylthioribulose-1-phosphate in methionine salvage. Assays contained no enzyme (black, negative control), 0.5 µg *B. thuringiensis* DrdA (red, positive control) or 25 µg of DEP1 (purple). Data are the mean of three replicates; error bars are the s.d. Where no error bars are evident, they are smaller than the filled circle symbol.

Supplementary Table 1. Primers used in this study

Primer	Name	Sequence	Application
Protein expression			pET28
1	BtKinN-XbaI-F	AGTC*TCTAGA*ATGTCTAAGTTCACAAAGTATTTTTTAATG	Kinase
2	BtKinN-XhoI-R	AGTC*CTCGAG*TTACTCCGCGTAGTGCATTG	
3	BtIsoN-NdeI-F	AGTC*CATATG*GAAGAGCAATTAATACCAATTC	Isomerase
4	BtIsoN-Xho-R	AGTC*CTCGAG*TTATTGAAATATCTTTTTTAAATTCTCCG	
5	BtAldo-C-PciI-F	GACT*ACATGT*TATTACAAAAAGAAAGAGAAG	Aldolase
6	BtAldo-C-XhoI-R	GACT*CTCGAG*ATACTGTTGCCCATACCC	
7	BtAldo-NcoI-F	GACT*CCATGG*GGATGTTATTACAAAAAGAAAGAGAAGAAAT	Tag-free aldolase
8	BtAldo-XhoI-R	GA*CTCGAG*TTAATACTGTTGCCCATACCCTTC	
9	At5G53850F-NcoI	AGTC*CCATGG*CGGTGGCTGCAG	Arabidopsis DEP1 (At5g53850)
10	At5G53850R-XhoI	AGTC*CTCGAG*GATTTGGGAGA ATGATGTGACAGTC	
Primers for *drdA* deletion construct			
11	SOE-IsoF	ATGATGGAAGAGCAATTAATACC	Isomerase SOEing
12	SOE-IsoR	CCTGCCTTTCCTCCCTCAAGTATGAAGCTTATGATGACTGAC	
13	SOE-BKEF	GTCAGTCATCATAAGCTTCATACTTGAGGGAGGAAAGGCAGG	BKE Selection Cassette
14	SOE-BKER	GAAACACCCTCAAGATTTTTTCCGCCGTATCTGTGCTCT	
15	SOE-SideroF	AGAGCACAGATACGGCGGAAAAAATCTTGAGGGTGTTTC	Siderophore (downstream gene) SOEing
16	SOE-SideroR	ACATTAATGGGACAAGGGAC	
17	SOE-IsoFXba	AGTC*TCTAGA*ATGATGGAAGAGCAATTAATACC	To clone into pDR244 XbaI site
18	SOE-SideroRXba	AGTC*TCTAGA*ACATTAATGGGACAAGGGAC	
Primers for *drdI* deletion construct			
19	SOEI-KinF	GCTTACAATTGGTGTGGAGG	Kinase SOEing
20	SOEI-KinR	CCTGCCTTTCCTCCCTCATTCTCCCCTTACTCCGC	
21	SOEI-BKEF	GCGGAGTAAGGGGAGAATGAGGGAGGAAAGGCAGG	BKE Selection Cassette
22	SOEI-BKER	CCTAAGTATGAAGCTTATGATGACTGCGCCGTATCTGTGCTCT	
23	SOEI-AldoF	AGAGCACAGATACGGCGCAGTCATCATAAGCTTCATACTTAGG	Aldolase/downstream gene SOEing
24	SOEI-MidSideroR	GCGTTGGGATTGAAATTG	
25	SOEI-All-F-XbaI	AGTC*TCTAGA*GCTTACAATTGGTGTGGAGG	To clone into pDR244 XbaI site
26	SOEI-All-R-XbaI	AGTC*TCTAGA*GCGTTGGGATTGAAATTG	
Primers for *drdA* deletion validation			
27	BKEOut-R	GCCTTTTCCTGAGCCGATTTC	Check for KO insertion orientation
28	IsoOut-F	CAATGCACTACGCGGAGTAAG	
29	BKE-OutF	GCATACATTATACGAACGGTAGAGAGAG	Check for KO insertion orientation
30	SideR-OutR	GTTGCCGCTATTGTCGCAATTG	
31	BtAldo-F	GGGACGGGTGGTAATATTAGTATC	Check for WT gene
32	BtAldo-R	GTTCCCCAATGCTTTTCGTTTG	
Primers for *drdI* knockout validation			
33	KinOut-F	GGAAGTATATTACGCTGATAGTTAGAAACATTG	Check for KO insertion orientation
34	MidSid-OutR	TCGATTAAACATTCTAGCATTAAACGAGC	Check for KO insertion orientation
35	BtIso-F	GTGGAAAGATGATGCTTTAGTTTTG	Check for WT gene
36	BtIso-R	ACTTTACTTTCTTTCGGAGCAG	

Supplementary Table 2. Data collection and refinement statistics (molecular replacement)

	DrdA (PDB: 6BTD)	DrdA+DHAP (PDB: 6BTG)
**Data collection**		
Space group	P 4 21 2	P 4 21 2
Cell dimensions		
a, *b*, *c* (Å)	103.724, 103.724, 48.817	105.104, 105.104, 49.366
α, β, γ (°)	90, 90, 90	90, 90, 90
Resolution (Å)	35.55-1.55 (1.61-1.55)*	35.98-1.70 (1.76 -1.70)
R_{sym} or R_{merge}	0.063 (0.51)	0.060 (0.571)
$I / \sigma I$	41.73 (7.37)	41.77 (6.74)
Completeness (%)	99.93 (99.29)	99.8 (98.4)
Redundancy	28.7 (28.3)	28.2 (27.9)
**Refinement**		
Resolution (Å)	1.55	1.70
No. reflections	1122770	874739
R_{work} / R_{free}	0.1584/0.1765	0.1677/0.1961
No. atoms		
Protein	1592	1627
Ligand/ion	6	11
Water	207	252
B-factors		
Protein	16.80	23.30
Ligand/ion	17.00	26.60
Water	33.90	40.00
R.m.s. deviations		
Bond lengths (Å)	0.008	0.009
Bond angles (°)	1.09	1.12

*Values in parentheses are for highest-resolution shell.
